# Removing barriers to COVID-19 vaccine intention in a university population: Results of a serial mediation study through the dimensions of the Health Belief Model

**DOI:** 10.1371/journal.pone.0322881

**Published:** 2025-05-16

**Authors:** Marine Paridans, Nadia Dardenne, Nicolas Gillain, Eddy Husson, Christelle Meuris, Gilles Darcis, Michel Moutschen, Claude Saegerman, Laurent Gillet, Fabrice Bureau, Anne-Françoise Donneau, Michèle Guillaume, Benoit Pétré

**Affiliations:** 1 Research unit Public Health: from Biostatistics to Health Promotion, University of Liège, Liège, Belgium; 2 Infectious Diseases Department, University Hospital of Liège, Liège, Belgium; 3 Fundamental and Applied Research for Animal and Health (FARAH) Centre, Liège University, Liège, Belgium; 4 Laboratory of Immunology-Vaccinology, FARAH, Liège University, Liège, Belgium; 5 Laboratory of Cellular and Molecular Immunology, GIGA Institute, Liège University, Liège, Belgium; The Chinese University of Hong Kong, HONG KONG

## Abstract

**Background:**

While many studies have used the Health Belief Model (HBM) to understand vaccine intention, none claim to have used serial mediation to understand the relationship between HBM dimensions and COVID-19 vaccine intention. This study developed a serial mediation model to assess the direct and indirect effects of the latent HBM dimensions on COVID-19 primary vaccine intention.

**Methods:**

A cross-sectional study: from 01 April to 10 June 2021, a self-administered online questionnaire on vaccine intention against COVID-19 was distributed to staff and students at the University of Liège (Belgium). Direct and indirect effects of the HBM dimensions (perceived susceptibility, severity, benefits, barriers, self-efficacy and cues to action) on vaccine intention (score 0–100) were assessed with serial mediation models. Actually, each permutation of the latent HBM dimensions, i.e., each causal chain, was assessed using partial least squares path modelling (PLS-PM) according to the order of the HBM dimensions in that particular chain.

**Results:**

The sample was made up of 1256 participants. The final model revealed that the causal chain with the lowest Bayesian Information Criterion (BIC) value was barriers (Effect estimation (CI95%): -0.09 (-0.15 - -0.03)) ↘ severity (-0.13 (-0.20 - -0.07)) ↘ low self-efficacy (0.20 (0.15–0.25)) ↘ low susceptibility (-0.55 (-0.60 - -0.51)) ↘ vaccine intention (outcome). This revealed a significant indirect and direct effect (-0.20 (-0.25 - -0.15)) between barriers and vaccine intention.

**Conclusions:**

The results demonstrated that perceived barriers are a key determinant in COVID-19 primary vaccine intention. Public health practitioners need to prioritise messaging that addresses the barriers reducing vaccine intention to enable individuals to make an informed choice. These messages could form part of a mass communication campaign aimed at hesitant individuals, with evidence-based information about vaccine safety a priority in order to establish a climate of trust.

## Introduction

Although the World Health Organization declared the end of the global public health emergency for the COVID-19 pandemic in May 2023 [1], it remains important to publish data collected during the pandemic, particularly on effective vaccination prevention strategies, to be better equipped to deal with future pandemics. As a reminder, the first vaccine against COVID-19 was licensed in Europe [2,3] at the end of 2020, opening the vaccination campaign. Vaccination has long been considered one of the most effective public health interventions for the prevention of infectious diseases [4], but it’s context must be linked to the intention to vaccinate. The COVID-19 pandemic and its particular context, such as rapid vaccine development and fear of potential side effects [5], fake news, misinformation and conspiracy theories [6] may have affected people’s negative vaccine intention. For this reason, understanding people’s vaccine intention and the reasons for them is crucial for developing appropriate messages that could lead to better informed choices being made about COVID-19 vaccination. A number of studies have highlighted the key factors influencing COVID-19 vaccination intention around the world but most studies have been carried out on the general population [7–15]. However, studying the factors influencing vaccine intention in specific groups such as the academic population is crucial if interventions tailored to the needs of this population are to be designed and developed [e.g., 10].

There are many different frameworks in the literature for investigating the potential determinants associated with vaccine intention. These include generic psychological theories that predict a wide variety of health-related behaviours, such as the Health Belief Model (HBM), the Theory of Planned Behaviour (TPB) [16] or more specific models that reflect predictors of vaccine intention and behaviour, such as the 5C model [17]. Among the psychological models, the HBM is one of the most widely used conceptual models and is a convenient framework for predicting vaccine intention. It has been used in many studies during previous pandemics [18–20], as well as during the COVID-19 pandemic [21]. In the context of COVID-19, the HBM has also been used to identify factors of acceptance of COVID-19 control measures [22]. These include six key dimensions that may influence vaccine intention, namely:

- perceived susceptibility (“*belief about the chances of experiencing a risk or getting a condition or disease”*);
- perceived severity *(“belief about how serious a condition and its sequelae are”*);
- benefits to action (*“belief in efficacy of the advised action to reduce risk or seriousness of impact”*);
- barriers to action (*“belief about the tangible and psychological costs of the advised action”*);
- cues to action (*“strategies to active readiness”)* and (6) self-efficacy (*“confidence in one’s ability to take action”*);
- self-efficacy (“*confidence in one’s ability to take action*”).

Other variables such as socio-demographic and psychological factors and knowledge are usually considered as covariates [23–25].

As mentioned above, although a large number of studies have already used the HBM to identify different factors associated with vaccine intention against COVID-19 or other diseases, relationships between the HBM dimensions seem to be undefined [25]. Indeed, in the majority of studies, the relationship between the outcome variable (i.e., COVID-19 vaccine intention or behaviour) and each of the main dimensions considered a covariate has been assessed without taking either the influence of the dimensions on each other into account [26,27] or other interactive effects [28]. On the other hand, some studies have used the HBM model while applying analytical approaches that take possible relationships between the dimensions of the HBM into account. These include mediation analyses [29]. For example, in Chen et al. 2021, one HBM dimension (self-efficacy) acted as a mediator between another HBM dimension (perceived barriers, perceived benefits and cues to action) and COVID-19 vaccine hesitancy [30]. In Jones et al. 2015, the authors noted the importance of investigating serial mediation, as the differential effects of HBM dimensions could be symptomatic of an underlying causal chain. Serial mediation has already been conducted on H1N1 influenza vaccine as a dependent variable and HBM dimensions as serial mediators [31], but not on COVID-19 vaccine intention and especially, not on an academic population.

If there is to be a better understanding of the importance and order of the different dimensions in health-related behaviour change in relation to vaccine intention against COVID-19 or other diseases in the future, it is crucial that the order of HBM dimensions in a causal chain be investigated. This will help to prioritise the organisation of public health messages in a COVID-19 or future epidemic vaccine programme and also provide additional information for vaccine crisis communication. For this reason, communicating messages that prioritise the factors with the greatest influence on vaccination intention will reduce vaccine hesitancy and enable individuals to make an informed decision about vaccinations.

The aim of this exploratory study was to develop a serial mediation model involving the six latent HBM dimensions, i.e., perceived susceptibility, perceived severity, benefits to action, barriers to action, cues to action and self-efficacy, and to assess how they directly and indirectly effected primary vaccine intention against COVID-19 in a university population.

## Materials and methods

### Context and study population

This research was part of a longitudinal study carried out among students and staff of the University of Liège (ULiège), Belgium, between April 2021 and December 2022, with the aim of studying SARS-CoV-2 infections, immune responses to SARS-CoV-2 infections and vaccines, and vaccine hesitancy (SARSSURV-ULiège study). The inclusion criteria were as follows: participants had to be between 18 and 67 years of age (67 being the legal retirement age in Belgium) and to give their participation consent via an online form. Any member of staff whose contract came to an end before 31 December 2021 and students (first year of Bachelor and diploma year) enrolled in the 2020–2021 academic year were excluded as the participants could not have been followed over a longer period of time [32]. To address this issue of vaccine hesitancy, several studies have been carried out [33,34] using the SARSSURV database, each with more specific objectives. This present study was a cross-sectional study carried out between April and June 2021.

The population of ULiège comprises 5633 staff and 28,064 students. All members of the university who met the study criteria were sent a personalised invitation by e-mail inviting them to participate in the SARSSURV study, i.e., 3576 staff and 25,378 students. This was a voluntary sampling method.

The inclusion criteria for this present study was:

- Registered in SARSSURV study between April and June 2021.

The exclusion criteria for this present study was:

- Prior vaccination against COVID-19.

Inclusion criteria were verified after data extraction by the principal investigator.

Several e-mail reminders inviting participation in the study minimised potential sources of non-response bias.

**Fig 1:**
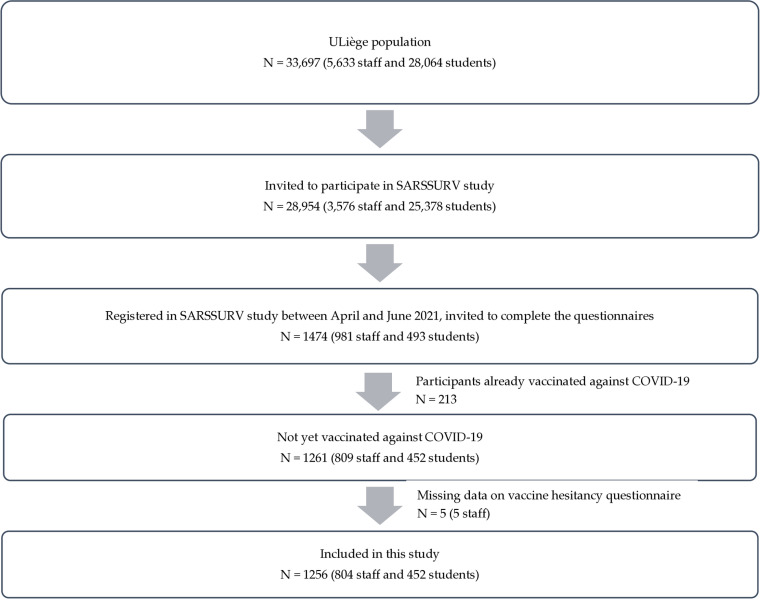
Flowchart of the study.

Between April and June 2021, 1474 participants (981 staff and 493 students) enrolled in SARSSURV study and were invited to complete the questionnaires on socio-demographic characteristics, medical characteristics and vaccine hesitancy. Of these participants, 213 had already been vaccinated and were therefore excluded from the study (because their vaccination could have influenced their responses to vaccine hesitancy). Missing data were identified for five staff members on the vaccine hesitancy questionnaire. Finally, a total of 1256 (804 staff and 452 students) were included in this study, as shown in Fig 1.

## Tools

The data were collected using self-administered questionnaires distributed through an online platform between 01 April and 10 June 2021.

As previously mentioned, the questionnaires were put together using a combination of models and measurement tools from the literature to determine the factors influencing vaccine hesitancy in general [17,35–37]. They were designed by members specialising in the field of research (infectious diseases, public health, etc.) and were adapted to the COVID-19 episode. The questionnaires were subsequently pre-tested by ten individuals. After the pre-test, a number of questions were clarified such as minor short-term side effects (e.g., pain, fatigue).

### Variables

#### Covariates.

##### Sociodemographic characteristics.

The socio-demographic characteristics included institutional status (student or staff member), gender (male or female), age (years), nationality (Belgian or other), province (Liège or other), faculty of work or study grouped into Medical Faculty (Faculty of Medicine and Faculty of Veterinary Medicine) or other, highest level of education grouped into high school and lower (no degree, primary, lower secondary, upper secondary), Bachelor degree (post-secondary non-tertiary education, short-type higher education) and University (university degree, PhD), and perceived socio-economic status grouped into upper, middle and working classes. In terms of social support, participants responded to two statements about social support in general, namely “People’s concern and interest in what you do?” (significant, moderate, difficult to determine, slight and none) and “How many people are close enough to you that you can rely on them when you have serious problems?” (no one - 1 or 2–3–5–6 or more) and the same question regarding social support since COVID-19.

##### Medical characteristics.

Body mass index (kg/m²), presence of a chronic disease (none - one or more) and past self-reported SARS-CoV-2 infection (yes/no) were collected. Chronic diseases included diabetes, hypertension, heart failure/coronary artery disease, history of stroke, liver failure/cirrhosis, kidney failure, chronic lung disease, asthma, autoimmune disease, immunodeficiency, hematologic or other cancer, organ or cell transplants, and other health problem(s).

##### General self-perception.

Each participant’s general self-perception was put together using four statements, namely “Compared to other people of my age, I am a careful person.”, “Compared to other people of my age, I am really careful about my health.”, “Compared to other people of my age, I am more likely to be sick.” and “Compared to other people of my age, I am an anxious person.” (Likert scale ranging from 0 (fully disagree) to 100 (fully agree)).

##### Health Literacy (HL).

The single item literacy screener (SILS), slightly modified for this study, was used in order to assess the health literacy of participants [38]: “When you read instructions, pamphlets, or other written material from your doctor or pharmacy, how often do you need help to understand the messages?” (Likert scale ranging from 0 (never) to 100 (always), the higher the score, the lower the literacy).

#### Health belief model.

##### Perceived (low) susceptibility.

Perceived (low) susceptibility was collected using the following two items: “COVID-19 is not serious enough for me to be vaccinated.” (Likert scale ranging from 0 (fully disagree) to 100 (fully agree)) and “My immune system is so strong that it protects me against COVID-19.” (Likert scale ranging from 0 (fully disagree) to 100 (fully agree) or don’t know; the higher the score, the lower the susceptibility).

##### Perceived severity.

One statement was used in order to assess perceived severity: “COVID-19 can seriously disrupt the life of a person affected by the virus” (Likert scale ranging from 0 (fully disagree) to 100 (fully agree) or don’t know; the higher the score, the higher the perceived severity).

##### Perceived benefits.

The perceived benefits of vaccination were collected using the following two statements: “The vaccine against COVID-19 provides better protection against the virus than the application of recommended barrier gestures.” (Likert scale ranging from 0 (fully disagree) to 100 (fully agree), the higher the score, the higher the perceived benefits) and “Contracting the disease provides better protection than the vaccine against COVID-19.” (Likert scale ranging from 0 (fully disagree) to 100 (fully agree) or don’t know; the higher the score, the lower the perceived benefits of vaccination). This item has been reversed in the mediation analysis to correspond to ‘the higher the score, the higher the perceived benefits of vaccination.”

##### Perceived barriers.

Four statements were used to assess perceived barriers: “There is a risk that I will develop minor short-term side effects after receiving the first and/or second dose of vaccine (pain, redness, fatigue, nausea, headache, etc.).” (Likert scale ranging from 0 (fully disagree) to 100 (fully agree)), “There is a risk that I will develop major short-term side effects after the first and/or second dose of vaccine.” (Likert scale ranging from 0 (fully disagree) to 100 (fully agree)) “There is a risk that I will develop long-term side effects after the vaccination.” (Likert scale ranging from 0 (fully disagree) to 100 (fully agree)) and “The fact that I will not be vaccinated by my own doctor is an obstacle to getting vaccinated.” (Likert scale ranging from 0 (fully disagree) to 100 (fully agree) or don’t know; the higher the score, the more important the barriers).

##### Cues to action.

One statement was used to assess specific internal or external indices: “How often do you have contact with people who are considered to be at risk of developing a severe form of COVID-19 (elderly person, person with chronic disease(s) such as diabetes, heart disease, respiratory disease, cancer, severe obesity, etc.)?” (At least once a week, once a month, 3 or 4 times a year, once a year or never).

##### Perceived (low) self-efficacy.

Information about perceived (low) self-efficacy was collected using the following statement: “The time it will take me to get vaccinated (travelling and waiting time included) is an obstacle to getting vaccinated.” (Likert scale ranging from 0 (fully disagree) to 100 (fully agree) or don’t know; the higher the score, the lower the self-efficacy).

#### Outcome.

##### COVID-19 primary vaccine intention.

Participants’ primary intention to be vaccinated against COVID-19 was assessed using the following item: “On a scale from 0 to 100, what is your current intention to be vaccinated when a vaccine is offered?” (Likert scale ranging from 0 (no intention) to 100 (total intention); the higher the score, the higher the intention).

### Statistical analysis

Results were expressed as numbers and frequencies for qualitative parameters and as median (P50) and interquartile range (IQR, P25-P75) for quantitative parameters because the normality of the distribution was not satisfied. In addition, non-parametric tests were used. The normality of the distribution of quantitative parameters was investigated using mean-median comparison, histogram and quantile-quantile plot, and tested using the Shapiro-Wilk hypothesis test.

For each item belonging to a dimension of the HBM model, a Spearman correlation coefficient (Rs) was calculated to assess the association between each statement and the responses, i.e., vaccine intention (score 0–100). Spearman correlation coefficients could be considered as very weak to weak if < 0.40, moderate if values varied from 0.40 to 0.60, strong from 0.60 to 0.80 and very strong if > 0.80 [39].

The association between each covariate (institutional status, gender, age, province, nationality, faculty, highest level of education, perceived socioeconomic status, BMI, chronic disease and self-reported past SARS-CoV-2 infection, HL, social support and general self-perception) and vaccine intention (score 0–100) was tested using the non-parametric Kruskal-Wallis test for qualitative covariates and by means of the Spearman correlation hypothesis test for quantitative covariates. The effect size eta-squared (η²) were calculated when results from the non-parametric Kruskal-Wallis test were found to be significant. The effect size was considered as small if 0.01 < η² < 0.06, median if 0.06 < η² < 0.14 and large if η² > 0.14 [40].

To evaluate the direct and indirect effects of the HBM latent dimensions on vaccine intention, serial mediation models for each permutation were assessed by means of structural equation modelling (SEM) using Partial Least Squares Path Modelling (PLS-PM) [41,42]. Given that the relationships between the dimensions of the HBM appeared undefined (see Introduction section), an attempt was made to explore all possible causal chains, without determining any prior order. The Bayesian Information Criterion (BIC) was used to compare the models. Internal consistency reliability was assessed using Rhoc and Cronbach’s alpha indices. In exploratory analyses, values between 0.60 and 0.70 for these indices were considered acceptable, while values between 0.70 and 0.90 were deemed satisfactory to good. Values above 0.90 indicated that the indicators were redundant [43]. Convergent and discriminant validity were estimated using the average variance extract (AVE) and the heterotrait-monotrait ratio (HTMT), respectively. For AVE, values had to be higher than 0.5 [44], while HTMT had to be less than 0.9 [45]. Effect sizes were evaluated using f-square. Values of 0.02, 0.15, and 0.35 were considered small, medium, and large effect sizes, respectively [46]. Significance of effect was tested by bootstrapping (1000 bootstrap subsamples). Confounding covariates such as sociodemographic variables, health literacy, general self-perception, body mass index, chronic disease and previous SARS-CoV-2 infection were included in the models.

All statistical analyses were performed globally and by institutional status, i.e., staff and students.

The proportion of “don’t know” responses received for statements in the HBM model varied from 1.43% to 58.1%. This implied a loss of almost 87% (1089/1256) of the data. A score of 50% was therefore used to replace these values to carry out the statistical analyses to avoid losing such a large percentage of the data. A sensitivity analysis was subsequently conducted to compare results with and without (n = 167) replacing these values.

The results were significant at the 5% critical level (p < 0.05). Statistical analyses were performed using the statistical package SAS (version 9.4 for Windows) and R (version 4.0) with specific packages seminR [47].

All analyses were carried out on all available data

### Ethical and legal aspects

The study was approved by the Ethics Committee of the University Hospital of Liège (reference number 2021/96, dated 26 March 2021). Written informed consent online was obtained from each participant before they were accepted. After enrolment on the SARSSURV study, a unique identification code (ID) was assigned to each participant [32]. All data was treated confidentially by the SARSSURV team and anonymised before any analysis was performed. Compliance with data protection regulations was approved by the official data protection officer of the University of Liège.

## Results

### Description of the data

The study included 1256 subjects, of whom 804 were staff (64%) and 452 were students (36%). The median vaccine intention score was 100 (IQR: 78–100) for the whole population and 100 (IQR: 80–100) and 98.5 (75–100) for staff and students respectively. The proportion of participants with 100% intention was equal to 51.5% (n = 647) for the whole population, 52.9% (n = 425) for staff and 49.1% (n = 222) for students. There were 60.3% women and 39.7% men and this distribution was similar for the two institutional statuses. The median age was 34.5 (IQR: 24.1–46.9) years for the whole sample, 43.3 (IQR: 34.9–51.3) years for staff and 22.7 (IQR: 21.0–25.6) years for students. Almost 3/4 of the participants had a normal BMI, regardless of their institutional status. Just over 20% of staff reported at least one previous SARS-CoV-2 infection, rising to almost 25% among students. All other covariates are presented in Table 1, globally and by institutional status. Vaccine intention scores were also reported for each status. For the whole population, gender, age, highest level of education, perceived socio-economic status, HL, and general self-perception were found to be statistically significantly associated with vaccine intention (p < 0.05), while statistically significant relationships were only observed between vaccine intention and, respectively, gender, age, BMI, HL and general self-perception for staff members; and highest level of education, living standards, HL and general self-perception for students (Table 1). Although these relationships could be considered significant, they presented small effect sizes (η² < 0.06, R_S_ < 0.40) (see Materials and Methods section).

**Table 1:** Characteristics of the 1256 participants included in the study and association with vaccine intention, globally and by institutional status.

		Globally (n = 1256)		Staff members (n = 804)		Students (n = 452)	
Variable	Categories	Number (%)	Vaccine intention P50 (P25 – P75)	Number (%)	Vaccine intention P50 (P25 – P75)	Number (%)	Vaccine intention P50 (P25 – P75)
Institutional status							
	Staff member	804 (64.0)	100 (80–100)				
	Student	452 (36.0)	98.5 (75–100)				
Gender							
	Female	757 (60.3)	99 (75–100)*	483 (60.1)	100(76–100)*	274 (60.6)	98.5 (75–100)
	Male	499 (39.7)	100 (81–100)* η² = 0.004	321 (39.9)	100 (85–100)* η² = 0.005	178 (39.4)	96.5 (70–100)
Age (years)	P50 (P25-P75)	34.5 (24.1–46.9)	RS = 0.090**	43.3 (34.9–51.3)	RS = 0.11**	22.7 (21–25.6)	R_S _= 0.016
Province							
	Liège	1062 (84.6)	100 (80–100)	696 (86.6)	100 (80–100)	366 (81.0)	98.5 (75–100)
	Other	194 (15.4)	97.5 (75–100)	108 (13.4)	99 (72.5–100)	86 (19.0)	99.5 (75–100)
Nationality							
	Belgian	1130 (90.0)	100 (80–100)	744 (92.5)	100 (80–100)	386 (85.4)	98.5 (75–100)
	Other	126 (10.0)	100 (73–100)	60 (7.5)	100 (73–100)	66 (14.6)	98 (75–100)
Faculty							
	Medical fields	258 (20.5)	100 (81–100)	130 (16.2)	100 (85–100)	128 (28.3)	98.5 (75–100)
	Other	998 (79.5)	100 (76–100)	674 (83.8)	100 (78–100)	324 (71.7)	100 (76–100)
Highest level of education							
	High school or lower	291 (23.2)	95 (70–100)**	33 (4.1)	95 (70–100)	258 (57.1)	98.5 (75–100)*
	Bachelor degree	259 (20.6)	95 (70–100)**	198 (24.6)	99 (75–100)	61 (13.5)	95 (72–100)**
	University	706 (56.2)	100 (80–100) η² = 0.011	573 (71.3)	100 (80–100)	133 (29.4)	90 (66–100) η² = 0.022
Perceived socio-economic status by class							p = 0.002
	High	124 (11.2)	90 (60–100)***	49 (6.8)	98 (76–100)	75 (19.2)	100 (75–100)*
	Middle	328 (29.6)	99.5 (70–100)*	220 (30.7)	97.5 (70–100)	108 (27.7)	85 (33–100)**
	Working	655 (59.2)	100 (85–100) η² = 0.011	448 (62.5)	100 (84.50–100)	207 (53.1)	100 (71.5–100) η² = 0.024
Body Mass Index (kg/m²)	P50 (P25 – P75)	23.3 (21.0–26.2)	RS = 0.051	23.8 (21.5–26.7)	RS = 0.11**	22.5 (20.4–25.1)	RS = -0.066
Chronic disease							
	No	987 (78.6)	100 (75–100)	608 (75.6)	100 (78–100)	379 (83.8)	97 (75–100)
	≥1	269 (21.4)	100 (81–100)	196 (24.4)	100 (81–100)	73 (16.2)	100 (80–100)
Previous self-reported SARS-CoV-2 infections							
	None	985 (78.4)	100 (78–100)	645 (80.2)	100 (80–100)	340 (75.2)	97.5 (75–100)
	≥1	271 (21.6)	100 (77–100)	159 (19.8)	98 (76–100)	112 (24.8)	100 (80–100)
Health literacy (0–100)	P50 (P25 – P75)	7.0 (0.0–20.0)	RS = -0.13***	6.00 (0.00–16.0)	RS = -0.084*	10.0 (0.00–20.0)	RS = -0.19***
Social support							
Number of relatives who could be counted on							
	None	7 (0.6)	77 (30–100)	5 (0.6)	90 (65–100)	2 (0.4)	53.5 (30–77)
	1-2	227 (18.1)	100 (80–100)	166 (20.6)	100 (80–100)	61 (13.5)	100 (70–100)
	3-5	612 (48.7)	100 (80–100)	385 (47.9)	100 (80–100)	227 (50.2)	98 (75–100)
	6+	410 (32.6)	99 (75–100)	248 (30.8)	99.5 (75–100)	162 (35.8)	95 (75–100)
Number of relatives who can be counted on since COVID-19							
	None	14 (1.1)	100 (80–100)	11 (1.4)	95 (67.00–100)	3 (0.7)	30 (20–77)
	1–2	278 (22.1)	100 (80–100)	196 (24.4)	100 (80–100)	82 (18.1)	100 (78–100)
	3–5	606 (48.2)	95 (75–100)	377 (46.9)	100 (81–100)	229 (50.7)	98 (75–100)
	6+	358 (28.5)	100 (78–100)	220 (27.4)	96.5 (73–100)	138 (30.5)	95 (75–100)
People’s concern and level of interest in what you do							
	Significant	414 (33.0)	100 (75–100)	267 (33.2)	100 (80–100)	147 (32.5)	100 (80–100)
	Moderate	484 (38.5)	95 (78–100)	324 (40.3)	100 (80–100)	160 (35.4)	90 (70–100)
	Difficult to determine	249 (19.8)	100 (75–100)	147 (18.3)	95 (77–100)	102 (22.6)	95 (80–100)
	Slight	99 (7.9)	100 (95–100)	58 (7.2)	100 (80–100)	41 (9.1)	98 (64–100)
	None	10 (0.8)	100 (78–100)	8 (1.0)	100 (97.5–100)	2 (0.4)	90 (80–100)
General self-perception	P50 (P25 – P75)						
I am a careful person		80 (70–90)	RS = 0.15***	80 (72–90)	RS = 0.15***	80 (70–90)	RS = 0.13**
I am a very health-conscious person		75 (60–85)	R_S_ = -0.0030	75 (60–85)	R_S_ = -0.028	73.5 (60–85)	R_S_ = 0.032
I am regularly sick		15 (4–30)	RS = -0.062*	10.5 (1–27)	R_S_ = -0.055	20 (8–40)	R_S_ = -0.058
I am anxious		50 (20–73.5)	R_S_ = 0.0029	50 (20–70)	R_S_ = -0.010	55 (23–76.5)	R_S_ = 0.027

*p < 0.05, ** p < 0.01, *** p < 0.0001

### The health belief model

Globally, the following dimensions of the HBM were found to have items that were significantly (p < 0.01) associated with vaccine intention, with Spearman correlation coefficients ranging in absolute value from 0.085 to 0.63: “perceived (low) susceptibility”; the higher the score, the lower the vaccine intention (R_S _= -0.63 and R_S_ = -0.37 corresponding respectively to strong and moderate correlation, p < 0.0001) and was the dimension that presented the strongest correlations with vaccine intention. The others were “perceived severity”; the higher the score, the higher the vaccine intention (R_S_ = 0.31, p < 0.0001), “perceived benefits”, the higher the score, the higher the vaccine intention(R_S_ = 0.25 and R_S_ = -0.25 respectively, p < 0.0001), “perceived barriers”; the higher the score, the lower the vaccine intention (R_S_ = 0.085, R_S_ = -0.23, R_S_ = -0.34, R_S_ = -0.28 respectively, p < 0.001) where the first item presented the weakest correlation with vaccine intention but was nevertheless significant. For “perceived (low) self-efficacy”; the higher the score, the lower the vaccine intention (R_S _= -0.26, p < 0.0001). Most of these correlations were considered to be weak (see Materials and Methods section). The items of the “cues to action” dimension did not show a significant association with vaccine intention (p > 0.05). Similar results were observed according to institutional status (Table 2).

**Table 2:** Health Belief model dimensions and association with vaccine intention, globally and by institutional status (n = 1256).

			Globally (n = 1256)		Staff members (n = 804)		Students (n = 452)	
		% don’t know	P50 (P25 – P75)	Association	P50 (P25 – P75)	Association	P50 (P25 – P75)	Association
Vaccine intention (0–100)	1256	0	100 (78–100)		100 (80–100)		98.5 (75–100)	
**Perceived (low) susceptibility**								
*1. COVID-19 is not serious enough for me to be vaccinated*	1190	5.25	0 (0–20)	RS = -0.63***	0 (0–20)	RS = -0.65**	0 (0–23)	RS = -0.61***
*2. My immune system is so strong that it protects me from COVID-19*	1083	13.8	10 (0–39)	RS = -0.37***	5 (0–30)	RS = 0.41***	20 (0–50)	RS = -0.31***
**Perceived severity**								
*1. COVID-19 can seriously disrupt the life of a person*	1236	1.59	100 (87–100)	RS = 0.31***	100 (90–100)	RS = 0.31***	100 (84–100)	RS = 0.32***
**Perceived benefits**								
*1. COVID-19 vaccine is more useful in protecting against the virus than the application of recommended barrier measures*	1075	14.4	64 (42–83)	RS = 0.25***	62 (45–85)	RS = 0.24***	65.5 (40–80)	RS = 0.27***
*2. Contracting the virus offers more protection than the COVID-19 vaccine*	812	35.5	25 (10–52)	RS = -0.25***	25 (10–50)	RS = -0.25***	26 (8–55)	RS = -0.25***
**Perceived barriers**								
*1.There is a risk that I will develop minor side effects in the short term after receiving the first and/or second dose of vaccine*	1162	7.48	90 (76–100)	RS = 0.085**	93 (77–100)	RS = 0.051	90 (75–100)	RS = 0.14**
*2. There is a risk that I will develop major side effects in the short term after the first and/or second dose of vaccine*	699	44.3	20 (5–73)	RS = -0.23***	22 (7–75)	RS = -0.23***	16.5 (5–70)	RS = – 0.23***
*3. There is a risk that I will develop long–term side effects after vaccination*	526	58.1	7 (0–50)	RS = -0.34***	9 (0–60)	RS = -0.23***	5 (0–29)	RS = -0.38***
*4. The fact that it is not my own GP who vaccinates me is a deterrent to getting vaccinated*	1242	1.11	0 (0–10)	RS = -0.28***	0 (0–10)	RS = -0.28***	0 (0–11)	RS = -0.24***
**Cues to action**	1256	0		p = 0.40		p = 0.24		p = 0.80
*1. How often do you have contact with people considered to be at risk of developing a severe form of COVID- 19 (elderly, people with chronic disease(s) such as diabetes, heart disease, respiratory disease, cancer, severe obesity, etc.)?*	at least 1x/week		453 (36.1)	100 (75–100)	296 (36.8)	100 (76–100)	157 (34.7)	98 (72–100)
	at least 1x/month		417 (33.2)	99 (80–100)	263 (32.7)	100 (80–100)	154 (34.1)	95.5 (75–100)
	at least 3or 4x/year		200 (15.9)	100 (80–100)	130 (16.2)	100 (85–100)	70 (15.5)	100 (70–100)
	at least 1x/year		67 (5.3)	98 (68–100)	44 (5.5)	98.5 (67.5–100)	23 (5.1)	90 (70–100)
	Never		119 (9.5)	100 (80–100)	71 (8.8)	9 (77–100)	48 (10.6)	100 (86–100)
**Perceived (low) self–efficacy**								
*1. The time it will take me to get vaccinated (including travelling and waiting time) is a barrier to getting vaccinated*	1238	1.43	0 (0–9)	RS = -0.26 ***	0 (0–5)	RS = -0.29 ***	0 (0–10)	RS = -0.26 ***

*p < 0.05, ** p < 0.01, *** p < 0.0001

### Serial mediation models

The decision was taken to omit the “Perceived benefits” dimension from this analysis due to low values for reliability and validity criteria (alpha, RhoC and RhoA < 0.6) (Table A1 in S1 Appendix). Additionally, the first item of the “Perceived barriers” dimension was removed to improve these criteria and doing so improved the values of alpha (from 0.45 to 0.54), RhoC (from 0.66 to 0.77) and AVE (from 0.40 to 0.53) (Table 3 and Table A1 in S1 Appendix). For the final model, acceptable and satisfactory values were found for alpha (≥ 0.5), RhoC (> 0.70), AVE (> 0.5) and RHOA (> 0.5). Appropriate values were also observed for loading (> 0.5). The same conclusions were therefore drawn for the whole population by institutional status (i.e., staff and students) (Table 3).

**Table 3:** Reliability and convergent validity for the final model, globally and by institutional status (n = 1256).

	Globally (n = 1256)					Staff members (n = 804)					Students (n = 452)				
	Alpha	RhoC	AVE	RHOA	Loading	Alpha	RhoC	AVE	RHOA	Loading	Alpha	RhoC	AVE	RHOA	Loading
**Perceived (low) susceptibility**	0.65	0.84	0.73	0.73		0.63	0.84	0.72	0.72		0.66	0.85	0.74	0.80	
*1. COVID-19 is not serious enough for me to be vaccinated*					0.92					0.91					0.93
*2. My immune system is so strong that it protects me from COVID-19*					0.79					0.79					0.78
**Perceived barriers**	0.54	0.77	0.53	0.59		0.55	0.77	0.55	0.62		0.50	0.75	0.51	0.54	
*2. There is a risk that I will develop major side effects in the short term after the first and/or second dose of vaccine*					0.76					0.79					0.72
*3. There is a risk that I will develop long-term side effects after vaccination*					0.86					0.88					0.85
*4. The fact that it is not my own GP who vaccinates me is a deterrent to getting vaccinated*					0.52					0.50					0.54

**Fig 2:**
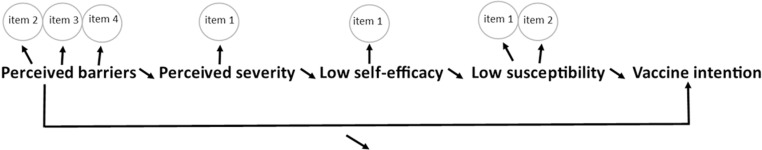
Final serial mediation model (n = 1256).

The “Cues to action” dimension was also left out of the final model because this dimension was not significantly associated with either the other dimensions or with vaccine intention (Table B4 in S1 Appendix). Non-significant covariates were also removed to improve BIC and R² criteria (Table B4 in S1 Appendix). Finally, and as illustrated in Fig 2, the final serial mediation model with the lower BIC and higher R² for the whole population (BIC = -592.65, R² = 0.40) and by institutional status (BIC = -394.10, R² = 0.42 for staff members; BIC = -177.69 and R² = 0.39 for students) was perceived barriers - perceived severity - perceived (low) self-efficacy - perceived (low) susceptibility - vaccine intention. From this model it was seen, for serial mediation effects, that participants who perceived more barriers perceived less severity (Estimation (CI95%): -0.092 (-0.15 - -0.027), f-square = 0.010), those who perceived less severity perceived less self-efficacy (Estimation (CI95%): -0.13 (-0.20 - -0.072), f-square = 0.020), those who perceived less self-efficacy perceived less susceptibility (Estimation (CI95%): 0.20 (0.15–0.25), f-square = 0.042), and, a direct effect, less perceived susceptibility was negatively related to vaccine intention (Estimation (CI95%): -0.55 (-0.60 - -0.51), f-square = 0.47). Perceived more barriers was, as a direct effect, negatively related to vaccine intention (Estimation (CI95%): -0.20 (-0.25 - -0.15), f-square = 0.063). Small effect sizes were observed for the serial mediation model (from 0.010 to 0.063), whereas a large effect size (f-square = 0.47 > 0.15) was obtained for the direct pathway perceived (low) susceptibility - vaccine intention. Values for HTMT were found to be acceptable (< 0.9) globally and by institutional status (Table 4).

**Table 4:** Results for the final model, globally and by institutional status (n = 1256).

	Globally		Staff members		Students	
	Estimation (CI95%)1*	Discriminant validity HTMT (CI95%)^1^*	Estimation (CI95%)2*	Discriminant validity HTMT (CI95%)2*	Estimation (CI95%)3*	Discriminant validity HTMT (CI95%)3*
**Serial mediation effect**						
Perceived barriers → Perceived severity	-0.092 (-0.15 – -0.027)	0.13 (0.077–0.20)	-0.074 (-0.17–0.002)	0.11 (0.070–0.21)	-0.13 (-0.21 – -0.062)	0.17 (0.095–0.28)
	f-square = 0.010		f-square = 0.093		f-square = 0.017	
Perceived severity → Perceived (low) self-efficacy	-0.13 (-0.20 – -0.072)	0.13 (0.072–0.20)	-0.16 (-0.25 – -0.080)	0.16 (0.080–0.25)	-0.085 (-0.18 – -0.016)	0.085 (0.017–0.18)
	f-square = 0.020		f-square = 0.027		f-square = 0.010	
Perceived (low) self-efficacy → Perceived (low) susceptibility	0.20 (0.15–0.25)	0.24 (0.18–0.30)	0.18 (0.096–0.24)	0.23 (0.13–0.30)	0.21 (0.13–0.31)	0.25 (0.15–0.37)
	f-square = 0.042		f-square = 0.084		f-square = 0.046	
**Direct effect**						
Perceived barriers → Vaccine intention	-0.20 (-0.25 – -0.15)	0.43 (0.37–0.48)	-0.21 (-0.26 – -0.14)	0.43 (0.34–0.50)	-0.20 (-0.28 – -0.13)	0.45 (0.35–0.57)
	f-square = 0.063		f-square = 0.057		f-square = 0.059	
Perceived (low) susceptibility → Vaccine intention	-0.55 (-0.60 – -0.51)	0.71 (0.66–0.77)	-0.56 (-0.64 – -0.50)	0.77 (0.67–0.82)	-0.51 (-0.61 – -0.44)	0.65 (0.56–0.76)
	f-square = 0.47		f-square = 0.52		f-square = 0.40	
**Total effect**						
Perceived barriers → Vaccine intention	-0.20 (-0.25 – -0.15)	0.43 (0.37–0.48)	-0.21 (-0.27 – -0.14)	0.43 (0.34–0.50)	-0.20 (-0.28 – -0.13)	0.45 (0.35–0.57)
Perceived severity → Vaccine intention	0.015 (0.008–0.025)	0.31 (0.24–0.38)	0.017 (0.005–0.028)	0.28 (0.17–0..35)	0.009 (0.002–0.024)	0.35 (0.26–0.47)
Perceived low self-efficacy → Vaccine intention	-0.11 (-0.14 – -0.078)	0.16 (0.10–0.23)	-0.10 (-0.14 – -0.06)	0.16 (0.081–0.24)	-0.11 (-0.17 – -0.061)	0.15 (0.030–0.27)
Perceived low susceptibility → Vaccine intention	-0.55 (-0.60 – -0.51)	0.71 (0.66–0.77)	-0.57 (-0.64 – -0.50)	0.77 (0.67–0.82	-0.51 (-0.61 – -0.44)	0.65 (0.56–0.76)
	BIC = -592.65 R² = 0.40		BIC = -394.10 R² = 0.42		BIC = -177.69 R² = 0.39	

1 Adjusted by gender, highest level of education, faculty and health literacy

2 Adjusted by gender, faculty and health literacy

³Adjusted by gender, health literacy, perceived socio-economic status and faculty

* Estimated by bootstrapping method

### Sensitivity analysis

A sensitivity analysis was performed to verify that the results obtained remained similar without replacing the “don’t know” values with a score of 50%. First, the associations between each dimension of the HBM and the vaccine intention score remained identical, with similar values for Spearman correlation coefficients (Table B1 in S1 Appendix). Secondly, the convergence criteria for reliability and validity, obtained from a final sample with a reduced number of 167 subjects, resulted in the same conclusions, the “perceived benefits” dimension and the same item for the “perceived barriers” dimension were removed for the same reasons as explained above. Furthermore, acceptable or even satisfactory values were found for the various quality indices (RhoC > 0.7, AVE > 0.5, RHOA ≥ 0.5), despite an alpha value of 0.48 for one HBM dimension (Table B2 in S1 Appendix). Thirdly, the results for the final model led to the same serial mediation model, with similar mediation, direct, with still a large effect size (f-square = 0.35) and total effects, as well as HTMT values that remained acceptable. However, the R² obtained was lower (R² = 0.35) (Table B3 in S1 Appendix).

## Discussion

This was the first study in Belgium to develop a serial mediation model to assess the direct and indirect effects of the six HBM dimensions (perceived (low) susceptibility, severity, benefits, barriers, (low) self-efficacy and cues to action) on COVID-19 primary vaccine intention in an academic population, namely staff and students at the University of Liège. This article provides a clearer understanding of how effective communication could improve COVID-19 vaccine intention, in a health emergency context where vaccination is perceived as a collective responsibility to protect vulnerable people, reduce hospitalizations and save lives.

### First analysis steps

Initial investigations confirmed that the HBM is a relevant model for predicting the determinants associated with vaccine intention against COVID-19. Indeed, statistically significant correlations were found between almost all dimensions (perceived barriers, benefits, severity, low susceptibility, low self-efficacy) and COVID-19 vaccine intention. These associations confirm the results of Limbu et al. 2023, a systematic study that demonstrated the HBM dimensions were most often associated with COVID-19 vaccine intention [21]. Beyond the HBM, these dimensions/results were also found in other studies on factors associated with COVID-19 vaccine hesitancy in general [e.g., 8–14]. Surprisingly, in the present study, the “Cues to action” dimension was not significantly associated with vaccine intention. This dimension was introduced using the statement “Contact with people considered to be at risk of developing a severe form of COVID-19”. However, an association between the “Cues to action” dimension and COVID-19 vaccine intention appeared when other items were used in studies of the general population or university students, such as COVID-19 vaccine recommendation from health care workers [48,49], general practitioner, leaders, friends and family, official guidelines from the Ministry of Health [26], sufficient information, social norm [50]. However, this information could not be included in this study. Another possible explanation for the non-association with the dimension could be that students may have been less interested in external factors that would encourage vaccination, such as campaigns and other information on COVID-19 vaccination. No difference in significant associated HBM dimensions was found according to institutional status. However, in the systematic review mentioned above, perceived severity/susceptibility and intention to vaccinate were less significantly associated with the student population [21]. Indeed, some studies conducted among university students, found that perceived severity [49,51] and susceptibility [49–51] were not significantly associated with vaccine intention. However, it should be noted that although the results obtained in this study were statistically significant, most of the correlation coefficients were < 0.40, concluding, except for perceived susceptibility, that the relationships between the items and vaccine intention were nevertheless poor [39]. This could explain the discrepancy with the literature. Another possible explanation is the timing of data collection, as participants’ beliefs possibly change over time.

From a public health perspective, these results indicated that the dimensions perceived barriers, benefits, severity, (low) susceptibility and (low) self-efficacy should be taken into consideration in public health messages to enable individuals to make an informed choice regarding vaccination against COVID-19. However, these results do not give the type messages that could be delivered as a priority.

### Serial mediation and its contributions

With serial mediation, the present study goes beyond the results of previous major studies by showing the direct and indirect effects of the HBM dimensions on COVID-19 vaccine intention and determining which dimension to focus public health messages on as a priority.

The final model was barriers - severity - (low) self-efficacy - (low) susceptibility - COVID-19 vaccine intention. This highlighted a significant indirect effect between barriers and vaccine intention through the other HBM dimensions. Overall, the causal chain showed that participants who perceived more barriers perceived less severity, and those who perceived less severity perceived less self-efficacy, who in turn perceived less susceptibility, and less perceived susceptibility was negatively related to vaccine intention. Thus, perceived barriers, namely the risk of developing side effects and the fact that the general practitioner did not vaccinate their patients, was the first dimension in the causal chain that influenced the other dimensions of the HBM in their associations with vaccine intention. A direct significant effect was also found between barriers and vaccine intention. This negative direct association between barriers and vaccine hesitancy/intention was found in [29,30], where SEM was also applied, although other statements were used in addition to side effects to assess perceived barriers, namely “Not enough research has been done about COVID-19 vaccine”, “The COVID-19 vaccine causes a person to get COVID-19”, “I am not sure if COVID-19 vaccine is effective in preventing the disease” [29,30]. Beyond the HBM, concerns about the side effects and safety of COVID-19 vaccines were identified as the main factors influencing students’ intention to vaccinate in a systematic review of the literature [52]. In addition, the direct pathway perceived (low) susceptibility - vaccine intention presented a large effect size (f-squared >0.15). This implies that even if perceived (low) susceptibility is not a priority factor in the causal chain, this dimension is still an important factor that influence vaccine intention.

From a public health perspective, these serial mediation results indicate that public health actors should prioritise messaging to address the barriers that reduce vaccine intention in order to enable individuals to make an informed choice. Our results offer a new perspective for discussing communication methodologies, such as those proposed by WHO [53], through message distribution. This use of messages to inform individuals could take place in mass mailing campaigns, where clear, evidence-based information about vaccine safety (without minimizing potential risks) could be communicated as a priority and a climate of trust could be established between the health authorities and the population. In a university environment such as ULiège, this information could be communicated by healthcare professionals/experts from various faculties (Medicine, Psychology, Social Sciences, etc.) and by students after reinforcing communication channels between universities and experts on the subject (scientists, public health experts, etc.) to ensure message consistency, or after reinforcing their knowledge via training or online courses. The introduction of online courses to increase university students’ knowledge of vaccines and immunisation has already proved its worth in other countries, such as Italy [54].

### Strengths of the study

It was possible to go further than previous major studies in order to understand the relationships between HBM dimensions and their effects on COVID-19 vaccine intention using a serial mediation model.

Although previous studies have used SEM to assess the effects of latent HBM dimensions [29,55], the mediation effects of these dimensions have been poorly documented. In particular, the mediation effects of the dimensions in relation to each other. For example, Adiyoso et al. 2023 examined the latent HBM dimension as a parallel mediator of the relationship between sociodemographic variables and vaccine acceptance, but failed to examine associations between the HBM dimensions in their model [55]. In Chen et al, the authors concluded that the HBM dimensions did not share parallel relationships but suggested that self-efficacy acted as a serial mediator. However, the authors did not examine serial mediation across all dimensions [30].

For exploratory purposes, this study considered all possible permutations to obtain the best causal chain from a statistical point of view, based on quality criteria such as BIC and R². The final model showed acceptable and satisfactory values for validity and reliability indices with alpha ≥ 0.5, RhoC > 0.70 [43], AVE > 0.5 [44] and RHOA > 0.5. Excellent values were also found for loading (> 0.5), while values for HTMT were found to be acceptable (< 0.9) [45]. These results were obtained for the whole population and by institutional status. Furthermore, the use of PLS-SEM was appropriate due to the non-normal data and in order to maximize the explained variance of the outcome variable [56].

Another strength was that university population, namely students and staff, provided data from a closed population, which in turn made it possible to address the factors that influence COVID-19 vaccine intention in a specific population.

### Study limitations

This study did, however, have several limitations. Firstly, the results may not be fully representative of a university population and cannot be representative of the Belgian population as a whole, because it was carried out on a voluntary sample from a highly educated university population with high health literacy and high intention to receive the COVID-19 vaccine. Indeed, several studies have shown an association between lower levels of health education and literacy and higher vaccine hesitancy [e.g., 8–11,15,57].

Also, there was a social desirability bias that may have influenced the results, although the questionnaire was administered to participants via an online platform to minimise this bias.

The data were self-reported by the participants and were not verified, particularly for medical characteristics, general self-perception, and social support.

One limitation of the study concerned the questionnaires used to measure COVID-19 vaccine intention, the factors influencing it and inclusion in the HBM model. Although these questionnaires were constructed on the basis of existing literature on vaccine hesitancy and adapted to the COVID-19 episode, they did not take into account the measurement models and tools proposed during the COVID-19 pandemic [58–62]. This limitation could be explained in particular by the absence of models and questionnaires more specific to the COVID-19 episode in the literature at the time the questionnaires were created.

Some items had a high percentage of “don’t know” responses (from 1.43% to 58.1%). As these items were scored from 0 to 100, in order not to consider these “don’t know” as missing data and to reduce the sample, these values were replaced by a score of 50%. A sensitivity analysis was performed and similar results were obtained even if the sample size was considerably reduced. However, as mentioned in [56], PLS-SEM can achieve results that reach high level of statistical power with smaller sample sizes. Despite the small sample size, values for correlation coefficient > 0.4 in absolute value and a large effect size (f-square = 0.35) for the direct pathway perceived (low) susceptibility - vaccine intention were observed*.* Therefore, the data replacement did not affect the conclusions.

Although results close to those in the literature were obtained for certain direct effects between the HBM dimensions and vaccine intention, the R² obtained was rather moderate [63], was lower than in Adiyoso et al. 2023 [55] (R² = 66.8%) or Berni et al. 2022 [29] (R² = 47.2%); this raises questions about the influence of the choice of items composing the different HBM dimensions and the associations between these dimensions.

Finally, although significant direct and indirect effects between the HBM dimension and vaccine intention were highlighted, the effect sizes were small for indirect effects (f-squared between 0.010 and 0.063). In contrast, the direct pathway perceived (low) susceptibility - vaccine intention presented a large effect size (f-squared >0.15). This implies that, even if indirect effects were highlighted, the vaccine intention was mainly explained by this relationship with perceived low susceptibility (> 0.15).

### Future research prospects

In terms of future research, similar studies need to be carried out on the general population or specific subgroups in order to develop appropriate public health messages. It might also be interesting to explore the relationships between the dimensions of other models such as TPB or other more integrative models (a combination of HBM and TPB for example). In addition, a longitudinal approach should be used, as beliefs about COVID-19 vaccines and COVID-19 vaccine intention may change over time [64–67].

## Conclusions

The results showed that the final mediation model was barriers - severity - (low) self-efficacy - (low) susceptibility - COVID-19 vaccine intention (outcome). This highlighted a significant indirect and direct effect between barriers and COVID-19 vaccine intention and showed that perceived barriers are a key determinant of COVID-19 primary vaccine intention among staff and students at the University of Liège, Belgium. Barriers to vaccination need to be prioritised in communication strategies to enable individuals to make informed choices. This prioritisation of message subject could be handled as mass communication campaigns, where clear, evidence-based information about vaccine safety could be communicated as a priority and a climate of trust established.

## Supporting information

S1 Appendix (DOCX)
