# Supplementary material for: Removing barriers to COVID-19 vaccine intention in a university population: Results of a serial mediation study through the dimensions of the Health Belief Model
Source: PLoS One. 2025 May 16;20(5):e0322881. doi: 10.1371/journal.pone.0322881 (PMC12083829; doi:10.1371/journal.pone.0322881)
Supplement: S1 Appendix — (DOCX) [file pone.0322881.s001.docx]

# SUPPORTING INFORMATION

**Reliability and Convergent validity**

| **Table A1. Reliability and convergent validity for different models** | | | | |
| --- | --- | --- | --- | --- |
|  | **With all items and with imputation**  **(n = 1256)** | | | |
|  | **Alpha** | **RhoC** | **AVE** | **RHOA** |
| **Perceived (low) susceptibility** | 0.65 | 0.84 | 0.73 | 0.74 |
| **Perceived benefits** | -0.21 | 0.0002 | 0.55 | 0.17 |
| **Perceived barriers** | 0.45 | 0.66 | 0.40 | 0.63 |

**Sensitivity analysis**

| **Table B1. Health Belief model dimensions and association with vaccine intention (n = 1256)** | | | | |
| --- | --- | --- | --- | --- |
|  | |  | **P50**  **(P25 – P75)** | **Association with vaccine intention** |
| Vaccine intention (0 – 100) | | 1256 | 100  (78 – 100) |  |
|  | |  |  |  |
| **Perceived (low) susceptibility** | |  |  |  |
| 1. COVID-19 is not serious enough for me to be vaccinated | | 1190 | 0  (0 – 20) | R_S_ = -0.61*** |
| 2. My immune system is so strong that it protects me from COVID-19 | | 1083 | 10  (0 – 39) | R_S_ = -0.37*** |
|  | |  |  |  |
| **Perceived severity** | |  |  |  |
| 1. COVID-19 can seriously disrupt the life of a person with this virus | | 1236 | 100  (87 – 100) | R_S_ = 0.31*** |
|  | |  |  |  |
| **Perceived benefits** | |  |  |  |
| 1. COVID-19 vaccine is more useful in protecting against the virus than the application of recommended barrier measures | | 1075 | 64  (42 – 83) | R_S_ = 0.25*** |
| 2. Getting sick is more protective than the COVID-19 vaccine | | 812 | 25  (10 – 52) | R_S_ = -0.29*** |
|  | |  |  |  |
| **Perceived barriers** | |  |  |  |
| 1. There is a risk that I will develop minor side effects in the short term after receiving the first and/or second dose of vaccine | | 1162 | 90  (76 – 100) | R_S_ = 0.072** |
| 2. There is a risk that I will develop major side effects in the short term after the first and/or second dose of vaccine | | 699 | 20  (5 – 73) | R_S_ = -0.28*** |
| 3. There is a risk that I will develop long-term side effects after vaccination | | 526 | 7  (0 – 50) | R_S_ = -0.46*** |
| 4. The fact that it is not my GP who vaccinates me is a deterrent to getting vaccinated | | 1242 | 0  (0 – 10) | R_S_ = -0.28*** |
|  | |  |  |  |
| **Cues to action** | 1256 | |  | p = 0.40 |
| 1. How often do you have contact with people considered to be at risk of developing a severe form of COVID-19 (elderly, people with chronic disease(s) such as diabetes, heart disease, respiratory disease, cancer, severe obesity, etc.)? | at least 1x/week | | 453 (36.1) | 100 (75 – 100) |
|  | at least 1x/month | | 417 (33.2) | 99 (80 – 100) |
|  | at least 3or 4x/year | | 200 (15.9) | 100 (80 – 100) |
|  | at least 1x/year | | 67 (5.3) | 98 (68 – 100) |
|  | Never | | 119 (9.5) | 100 (80 – 100) |
|  |  | |  |  |
| **Perceived (low) self-efficacy** |  | |  |  |
| 1. The time it will take me to get vaccinated (including travel and waiting time) is a barrier to getting vaccinated | 1238 | | 0  (0 – 0) | R_S_ = -0.25*** |
|  |  | |  |  |

**p < 0.01

*** p < 0.0001

**Table B2. Reliability and convergent validity for different models**

|  | **With all items and without imputation (n = 167)** | | | | **Final model without imputation**  **(n = 167)** | | | |
| --- | --- | --- | --- | --- | --- | --- | --- | --- |
|  | **Alpha** | **RhoC** | **AVE** | **RHOA** | **Alpha** | **RhoC** | **AVE** | **RHOA** |
| **Perceived (low) susceptibility** | 0.65 | 0.85 | 0.74 | 0.74 | 0.65 | 0.85 | 0.74 | 0.73 |
| **Perceived benefits** | -0.13 | 0.0 | 0.53 | 0.11 | / | / | / | / |
| **Perceived barriers** | 0.45 | 0.67 | 0.39 | 0.58 | 0.48 | 0.75 | 0.50 | 0.52 |

| **Table B3. Results for the final model (n = 167)** | | | |
| --- | --- | --- | --- |
|  |  | **Estimation**  **(CI95%)*** | **Discriminant validity HTMT (CI95%)*** |
| **Serial mediation effect** | |  |  |
| Perceived barriers 🡪 Perceived severity | | -0.071  (-0.12 – -0.002) | 0.11  (0.077 – 0.18) |
|  | | f-square = 0.005 |  |
| Perceived severity 🡪 perceived (low) self-efficacy | | -0.11  (-0.16 – -0.056)  f-square = 0.012 | 0.11  (0.056 – 0.16) |
| Low self-efficacy 🡪 perceived (low) susceptibility | | 0.17  (0.13 – 0.23)  f-square = 0.031 | 0.21  (0.15 – 0.28) |
| **Direct effect** | |  |  |
| Perceived barriers 🡪 Vaccine intention | | -0.22  (-0.27 – -0.17)  f-square = 0.071 | 0.45  (0.37 – 0.52) |
| Perceived (low) susceptibility 🡪 Vaccine intention | | -0.49  (-0.54 – -0.44)  f-square = 0.35 | 0.64  (0.59 – 0.70) |
| **Total effect** | |  |  |
| Perceived barriers 🡪 Vaccine intention | | -0.22  (-0.27 – -0.17) | 0.43  (0.37 – 0.48) |
| Perceived severity 🡪 Vaccine intention | | 0.009  (0.004 – 0.016) | 0.27  (0.22 – 0.38) |
| Perceived (low) self-efficacy 🡪 Vaccine intention | | -0.086  (-0.12 – -0.061) | 0.15  (0.10 – 0.21) |
| Perceived (low) susceptibility 🡪 Vaccine intention | | -0.49  (-0.54 – -0.44) | 0.64  (0.59 – 0.70) |
|  |  | BIC = -487.82 |  |
|  |  | R² = 0.35 |  |

| Table B4. Comparison of results for the different models | | | | | | |
| --- | --- | --- | --- | --- | --- | --- |
|  | Model with  the 6 HBM dimension |  | Model with  5 HBM dimensions |  | Model with  4 HBM dimensions | Model with  4 HBM dimensions  and covariates |
| Serial mediation effect | Estimation (ci95%) |  | Estimation (ci95%) |  | Estimation (ci95%) | Estimation (ci95%) |
| Perceived (low) susceptibility →  Cue to actions | 0.026  (0.006 – 0.093) | Perceived (low) susceptibility →  Cue to actions | 0.020  (-0.026 – 0.075) | Perceived barriers →  Perceived severity | -0.090  (-0.15 - -0.023) | -0.092  (-0.15 - -0.028) |
| Cue to actions →  Perceived (low) self-efficacy | 0.069  (0.003 – 0.12) | Cue to actions →  Perceived (low) self-efficacy | 0.069  (-0.002 – 0.12) | Perceived severity →  Perceived (low) self-efficacy | -0.13  (-0.20 - -0.072) | -0.13  (-0.20 - -0.072) |
| Perceived (low) self-efficacy → Perceived severity | 0.13  (0.072 – 0.20) | Perceived (low) self-efficacy → Perceived severity | -0.13  (-0.20 – -0.072) | Perceived (low) self-efficacy →  Perceived (low) susceptibility | 0.20  (0.15 – 0.25) | 0.20  (0.15 – 0.25) |
| Perceived severity →  Perceived benefits | -0.16  (-0.22 – 0.18) | Perceived severity →  Perceived barriers | -0.090  (-0.15 – -0.023) |  |  |  |
| Perceived benefits →  Perceived barriers | 0.19  (-0.24 – 0.24) |  |  |  |  |  |
| Direct effect |  |  |  |  |  |  |
| Perceived (low) susceptibility → Vaccine intention | -0.55  (-0.61 - -0.52) | Perceived (low) susceptibility → Vaccine intention | -0.55  (-0.61 - -0.52) | Perceived barriers→  Vaccine intention | -0.21  (-0.26 - -0.16) | -0.20  (-0.25 - -0.16) |
| Perceived barriers -→  Vaccine intention | -0.21  (-0.26 - -0.16) | Perceived barriers →  Vaccine intention | -0.21  (-0.26 - -0.16) | Perceived (low) susceptibility →Vaccine intention | -0.55  (-0.60 - -0.52) | -0.54  (-0.60 - -0.51) |
| Total effect |  |  |  |  |  |  |
| Perceived (low) susceptibility → Vaccine intention | -0.55  (-0.61 - -0.52) |  | -0.55  (-0.61 - -0.52) |  | -0.54  (-0.60 - -0.51) | -0.54  (-0.60 - -0.51) |
| Perceived severity → Vaccine intention | 0.006  (0.004 – 0.10) |  | 0.019  (0.005 – 0.034) |  | 0.015  (0.008- -0.25) | 0.015  (0.008- -0.25) |
| Perceived benefits → Vaccine intention | -0.039  (-0.051 – 0.052) |  |  |  |  |  |
| Perceived barriers → Vaccine intention | -0.21  (-0.26 - -0.16) |  | -0.21  (-0.26 - -0.16) |  | -0.21  (-0.26 - -0.16) | -0.20  (-0.25 - -0.16) |
| Perceived (low) self-efficacy → Vaccine intention | -0.001  (-0.002 - -0.0001) |  | -0.003  (-0.006 - -0.0001) |  | -0.11  (-0.15 - -0.079) | -0.11  (-0.15 - -0.079) |
| Cue to action →  Vaccine intention | 0 (0 – 0) |  | 0 |  |  |  |
|  | Bic = -612.86 |  | Bic = -612.30 |  | Bic = -610.59 | Bic = -530.66 |
|  | R² = 0.396 |  | R² = 0.396 |  | R² = 0.395 | R² = 0.40 |
